# *In Vitro* and *in Vivo* Evaluation of Novel Cross-Linked Saccharide Based Polymers as Bile Acid Sequestrants

**DOI:** 10.3390/molecules20033716

**Published:** 2015-02-24

**Authors:** Francisco Javier Lopez-Jaramillo, Maria Dolores Giron-Gonzalez, Rafael Salto-Gonzalez, Fernando Hernandez-Mateo, Francisco Santoyo-Gonzalez

**Affiliations:** 1 Departamento de Química Orgánica, Facultad de Ciencias, Instituto de Biotecnología, Universidad de Granada, E18071 Granada, Spain; E-Mail: fhmateo@ugr.es; 2 Departamento de Bioquímica y Biología Molecular II, Facultad de Farmacia, Universidad de Granada, E18071 Granada, Spain; E-Mails: mgiron@ugr.es (M.D.G.-G.); rsalto@ugr.es (R.S.-G.)

**Keywords:** cross-linked saccharides, hypercholesterolemia, bile acid sequestrants, cyclodextrin, dextrin, starch, divinyl sulfone

## Abstract

Bile acid sequestrants (BAS) represent a therapeutic approach for the management of hypercholesterolemia that relies on the cationic polymeric nature of BAS to selectively bind negatively charged bile acids. We hypothesized that the cross-linking of β-cyclodextrin (β-CD) and saccharides such as starch or dextrin with divinyl sulfone (DVS) yields homo- and hetero-polymeric materials with the ability to trap sterols. Our hypothesis was put to test by synthesizing a library of 22 polymers that were screened to evaluate their capability to sequester both cholesterol (CHOL) and cholic and deoxycholic acids (CA and DCA). Three polymers synthesized in high yield were identified as promising. Two were neutral hetero-polymers of β-CD and starch or dextrin and the third was a weakly cationic homo-polymer of starch, highlighting the importance of the cavity effect. They were tested in hypercholesterolemic male Wistar rats and their ability to regulate hypercholesterolemia was similar to that for the reference BAS cholestyramine, but with two additional advantages: (i) they normalized the TG level and (ii) they did not increase the creatinine level. Neither hepatotoxicity nor kidney injury was detected, further supporting them as therapeutical candidates to manage hypercholesterolemia.

## 1. Introduction

Cardiovascular diseases (CVD) are the leading cause of death in developed countries [1]. The root of clinical practice guidelines for these diseases is based on the definition of reference values and the measurement of traditional risk factors that, however, have been redefined and set lower over time. Hypercholesterolemia, an elevation of the total cholesterol (CHOL) and/or low-density lipoproteins (LDL) in the blood, is one such risk factor. This disorder leads to the accumulation of atheromatous plaques that is especially relevant when it takes place on the coronary arteries, narrowing them and reducing the blood flow supplied to the myocardium. The management of hypercholesterolemia is addressed by non-pharmacological approaches involving the modification of some risk factors, such as unhealthy diet, physical inactivity and tobacco, which very frequently need to be accompanied by pharmacological therapy [2]. The therapeutic approaches can be divided into statin and non-statin strategies [3,4]. Statins are competitive inhibitors of 3-hydroxy-3-methyl-glutaryl-CoA reductase (HMGCoA-R), a key rate-limiting enzyme of the CHOL biosynthetic pathway and they are the first-line pharmacological therapy because the biosynthesis of CHOL accounts for up to two-thirds of the CHOL pool of the body [5]. Non-statin drugs comprise niacin, fibrates, bile acid sequestrants (BAS) and natural extracts, being their nature and mechanisms of action diverse [5]. 

BAS exploits the fact that nearly 50% of the CHOL catabolism is governed by its conversion into bile acids (BA) to disrupt the reabsorption and favor their excretion, which in normal conditions is limited to about 5% [6,7]. In general, BAS are cationic polymeric hydrogels that selectively binds the negatively charged BA. However, fibers from daily diet show some capability of binding BA and polysaccharides have been used as starting materials to produce BAS due to their hydrophilicity, low toxicity and biocompatibility [8]. Thus, it has been reported the ability to sequester BA of dextran cross-linked with epichlorohydrin [9,10], methylans [11] or chitosan [12,13] functionalized with alkylammonium groups.

The ability of cyclodextrins (CDs) to form inclusion complexes is old knowledge and has found pharmaceutical applications [14,15]. In the context of BAS, the therapeutic potential of CDs to balance the CHOL homeostasis has been explored in animal models [16,17,18,19,20,21]. Thus, mice injected with [^14^C] chenodeoxycholate and [^3^H]cholate showed increased excretion of [^14^C] chenodeoxycholate in feces when they were fed with β-CD, whereas the effect of α-CD or γ-CD was neutral. However, cholestyramine, an anion-exchange resin currently used in hypercholesterolemia therapy as BAS, increased the radioactivity of both [^14^C] chenodeoxycholate and [3H]cholate [22]. Further studies have demonstrated that the ingestion of β-CD enhances the BA excretion without provoking depletion of lipophilic vitamins [16] and that the primary mechanism of the lipid lowering potential of β-CD seems to be consequence of its sterol binding capacity that leads the impaired reabsorption of circulating BA and intestinal adsorption [17]. Interestingly, α-CD has been demonstrated to reduce the absorption of saturated fatty acids from the diet, which promote the hepatic synthesis of CHOL and reduce its clearance, with the net effect of reducing the total CHOL in blood. This result paved the way for ongoing clinical trial in human [23].

CD-based polymeric materials are interesting by their biomedical and pharmaceutical applications [24]. They have been synthesized by cross-linking with epichlorohydrin, diepoxide, diisocyanate or anhydride mediated cross-linkers. In addition, these host materials have been prepared by combining polymers and CDs as for example the copolymerization of vinyl or (meth)acryloyl-modified CD monomers with vinyl monomers (acrylic acid, 2-hydroxyethyl methacrylate or N-isopropylacrylamide) [24]. More recently, citric acid has been used as a cross-linker for the synthesis of drug delivery polymers [25,26,27,28] and in a previous contribution of this volume we reported the synthesis of divinyl sulfone (DVS) cross-linked CD-based polymeric materials and their applications as sorbents and encapsulating agents [29].

Despite the potential of β-CD as a BAS and the wide range of options to polymerize it, to the best of our knowledge there is only a single report on the application of insoluble β-CD polymers to the sorption of bile salts [30]. In this work we explore the applications of the DVS cross-linked-based polymers [29] as BAS. We hypothesize that the homo- and hetero-polymeric materials obtained by the cross-linking of either β-CD and/or polysaccharides such as starch or dextrin with DVS should also have the ability to trap sterols and that those matrices may be of help to manage hypercholesterolemia.

## 2. Results and Discussion

The elevation of total CHOL and/or LD in blood (*i.e.*, hypercholesterolemia) is a risk factor in CVD. CHOL metabolism in humans shows two important features from the perspective of the design of compounds to manage hypercholesterolemia: (i) dietary CHOL is the only alternative to biosynthesis as source of CHOL in mammals, representing about 35% of the CHOL in plasma [31] and (ii) about 50% of the CHOL catabolism is directed to its conversion into BA [6,32]. Thus, compounds with the ability to sequester both BA and dietary CHOL are potential drug candidates to lower CHOL levels. In this context, the ability of CD to form inclusion complexes with CHOL and BA has not been exploited, despite it is old knowledge and animal models have demonstrated that the intake of β-CD enhances BA excretion and lowers TG levels [16,17,18,19,20]. To the best of our knowledge, there is a single report on the application of insoluble β-CD polymers to the sorption of bile salts [30], but since they were functionalized with alkyl quaternary ammonium groups to yield cationic materials as the classical BAS drugs cholestyramine, colestipol, colesevelam, colextran or colestilan [8], the ability of β-CD to form inclusion complexes cannot be accounted for by their mechanism of action. These facts led us to hypothesize that the cross-linking β-CD may yield insoluble polymeric materials with ability to trap both dietary CHOL and BA that may help in the management of hypercholesterolemia. 

In order to put to test our hypothesis, a library of 22 insoluble saccharide-based polymers was synthesized by cross-linking either β-CD and/or polysaccharides, such as starch or dextrin, with DVS. The selection of DVS as a cross-linker was based on its good hydrosolubility, allowing the synthesis of the polymers in water, as well as its reactivity as an excellent Michael acceptor, consequence of the sulfone’s electron withdrawing capability that makes it a good electrophile [33,34]. The library was designed to evaluate both the cavity effect and the ionic effect. The former allows the formation of inclusion complexes with apolar molecules whereas the latter promotes the electrostatic interactions with charged molecules. Thus the library consists not only of cross-linked β-CD polymers but also dextrin and starch homo-polymers as well as β-CD/dextrin and β-CD/starch hetero-polymers, and some of them were functionalized with two aliphatic amines (diethylamine and bis(2-hydroxyethyl)amine) or with one aromatic amine (bis(2-pyridylmethyl)amine).

**Figure 1:**
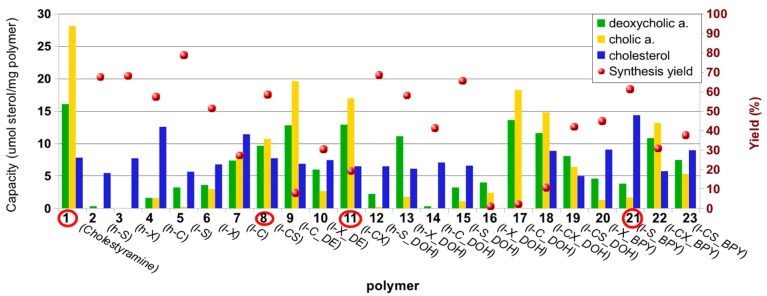
Screening against a solution of cholesterol (CHOL) and a solution of cholic and deoxycholic acids (CA and DCA) of the library prepared by cross-linking of β-CD (C), dextrin (X), starch (S) with a high (h) or low (l) DVS:saccharide stoichiometry and eventually with bis(2-pyridylmethyl)amine (BPY), bis(2-hydroxyethyl)amine (DOH) or diethylamine (DE). Each polymer is defined by a number and in brackets a summary of its composition. Units of the primary y-axes are μmoles of CHOL, CA and DCA per gram of polymer and % yield. Polymers encircled were assayed *in vivo* on hypercholesterolemic Wistar rats.

The library was screened against a solution of CHOL and a solution of cholic acid (CA) and dexoxycholic acid (DCA) to evaluate the ability of the polymers to sequester both diet CHOL and BA. At this point, it is important to recall that the solution of CA-DCA is a good model system because CA is the most abundant in bile, being its relative abundance 37.5% (25% for DCA), and the affinity of β-CD for them is among the lowest for the BA [22]. In fact, animal experimentation has revealed that the ingestion of β-CD promotes the excretion of chenodeoxycholate and decreases the percentage of taurochenodeoxycholate in biliary bile acids by −75% [17,22]. The result of the screening is summarized in Figure 1 and reveals some general trend. First, compared to cholestyramine (sample **1**), a reference ion-exchange resin currently used in hypercholesterolemia therapy as BAS, polymers show lower performance as expected considering the reported poor affinity of β-CD for CA [22,35]. Second, for homo-polymers (samples **2**–**7**) β-CD yields the best material regardless of the degree of cross-linking (samples **4** and **7**). In general, those polymers with lower degree of cross-linking show better sorption for BA than their high cross-linked counterparts (samples **2**–**4** *vs.* **5**–**7**). Hetero-polymers also show a good performance (samples **8** and **11**) with even higher ability to trap the BA. Third, the functionalization of the polymers with amines to favor electrostatic interactions with BA did not produce a clear general pattern. For the particular case of low cross-linked β-CD polymers, the functionalization of the homo-polymer (sample **7**) with aliphatic amines (samples **9** and **17**) yields an increase in the sorption of CA-DCA in detriment of CHOL, whereas for hetero-polymers (samples **8** and **11**), the functionalization with a aliphatic amines (samples **19** and **18**) or with the aromatic amine (samples **23** and **22**) does not present any remarkable advantage.

Some polymers show features that make them good candidates for animal experimentation. Polymers **9**, **11** and **17** share about 70% of the capacity of cholestyramine to sequester both CA and DCA (30-32 µmol/g *vs.* 44 µmol/g) pointing them out as potential BAS drugs. Additionally polymers **4** and **21** preferentially bind CHOL and may be potential food additives to reduce the absorption of CHOL. This may be an important approach in patients with altered CHOL homeostasis by increased CHOL absorption since they may not benefit from statin treatment, but may actually increase their cardiovascular risk when treated with drugs that reduce endogenous cholesterol synthesis [36]. Additionally, polymers **8** and **9** show similar capacity for CHOL and BA and may constitute a third category of attractive compounds that act simultaneously on the absorption of dietary CHOL and on the excretion of BA. However, the ability to sequester BA and CHOL is not the only factor to consider, since the yield of the synthesis is a critical parameter to take into account. Thus, for each group only the polymer that was synthesized with the highest yield was selected for animal experimentations: polymer **11** (19.4% yield *vs.* 7.6% and 2.2%) from the group of potential BAS, polymer **21** (61.3% yield *vs.* 57.4%) from the group of potential food additive, and polymer **8** (58.21% yield *vs.* 7.9%) from the third category.

Polymers **8** and **11** are neutral hetero-polymers of β-CD and starch or dextrin and their good performance can be justified taking into account the ability of CD to sequester both CHOL from the diet and BA [37,38]. However, the rationalization of the results obtained with polymer **21**, a charged homo-polymer of starch bearing bis(2-pyridylmethyl)amine, may not be so straightforward. It is well established that amylose, one of the two components of starch, forms helical structures with a central cavity that can accommodate guest molecules [39], having been proposed to act as binding sites for BA [22].

The synthesis of the above polymers was scaled up and unreacted components were eliminated by Soxhlet extraction with ethyl acetate. After drying in a vacuum oven, polymers were characterized. Elemental analysis (Table 1) revealed a similar percentage of sulfur, suggesting a comparable degree of cross-linking. The content of nitrogen supports the idea that compared to cholestyramine polymer **21** is a weak cationic material and that polymers **8** and **11** are neutral. 

**Table 1:** Elemental analysis of the polymers selected [**8** (l-CS), **11** (l-CX) and **21** (l-S_BPY); l = low DVS:saccharide stoichiometry, C = β-CD, X = dextrin, = starch; BPY = bis(2-hydroxyethyl)amine] for animal experimentation.

Compound	% N	% C	% S
Cholestyramine	5.6 ± 0.11	63.4 ± 0.15	-
Polymer **8**	-	39.9 ± 0.05	9.5 ± 0.11
Polymer **11**	-	40.3 ± 0.14	8.6 ± 0.03
Polymer **21**	0.4 ± 0.06	41.1 ± 0.00	8.5 ± 0.00

**Figure 2:**
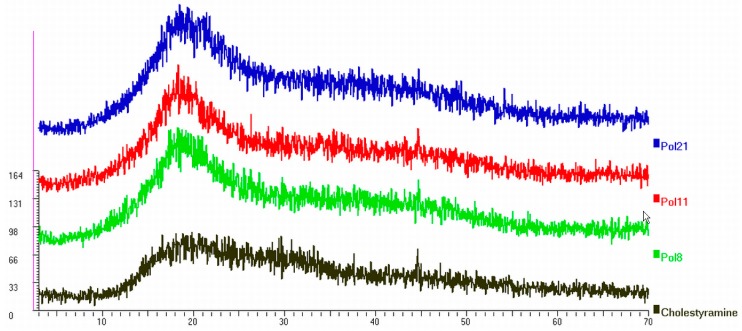
X-ray diffraction patterns of the polymers selected [**8** (l-CS), **11** (l-CX) and **21** (l-S_BPY); l = low DVS:saccharide stoichiometry, C = β-CD, X = dextrin, = starch; BPY = bis(2-hydroxyethyl)amine] for animal experimentation.

Powder X-ray diffraction patterns show a broad hump at 2θ = 13°–23° that indicates that the polymers, as well as cholestyramine, exist in a form of non-crystalline state (Figure 2).

**Figure 3:**
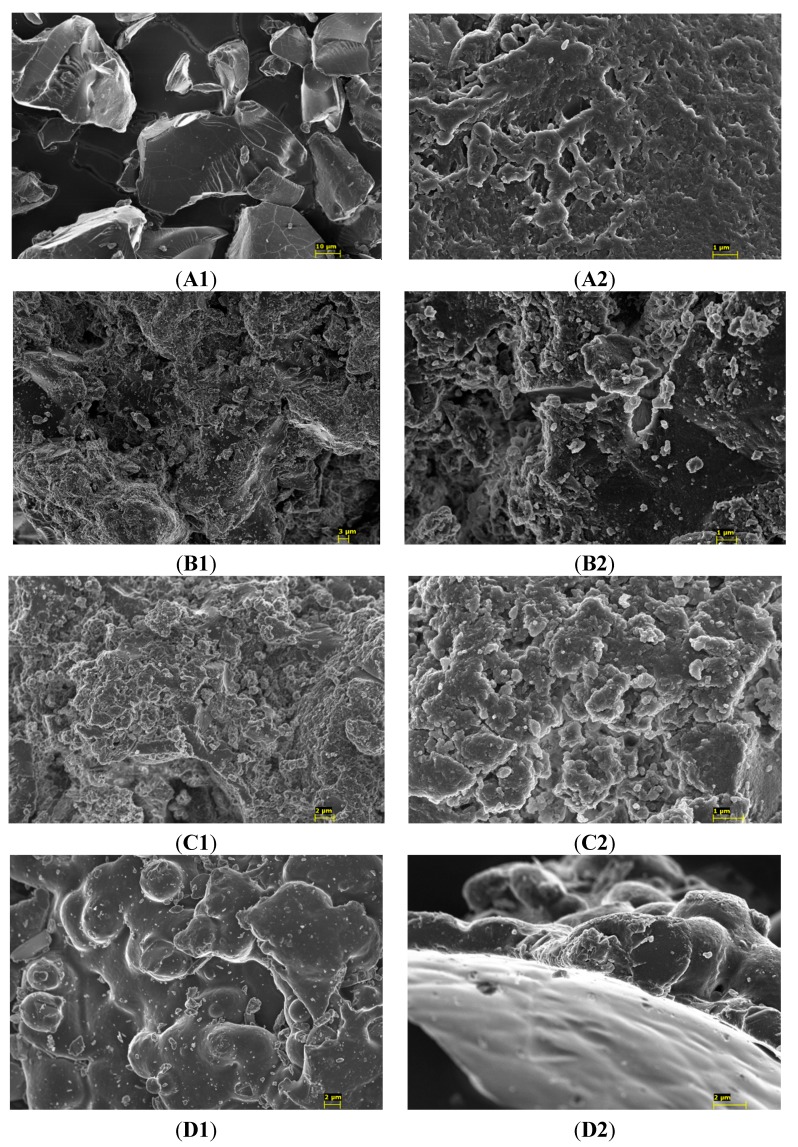
Electron microscopy analysis. Low (**left**) and high (**right**) resolution micrographs of the cationic polymers cholestyramine (**A**), polymer **21** (**B**), and neutral polymers **8** (**C**) and **11** (**D**), being the magnification ×2000 (**A1**), ×20,000 (**A2**), ×2500 (**B1**), ×15,000 (**B2**), ×7500 (**C1**), ×24,000 (**C2**), ×6000 (**D1**), ×13,000 (**D2**).

SEM images (Figure 3) reveals that the surface morphology is characteristic for each sample. Cholestyramine exhibits a rather homogeneous surface compared to cross-linked polymers and the presence of starch or dextrin seems to have a direct influence on the surface, those containing starch (B and C) being more irregular than that with dextrin (D *vs.* C).

*In vivo* analysis of cholestyramine and polymers **8**, **11** and **21** was carried out on hypercholesterolemic male Wistar rats. Hypercholesterolemia was induced by supplementing conventional diet with a 2% (w/w) CHOL (hypercholesterolemic diet) for two weeks. Then animals were fed with the hypercholesterolemic diet supplemented with 5% (w/w) of the polymer to be assayed. During the experiment, the weight of the animals increased up to a 170% during the first two weeks and then an additional 130% during the last two weeks, no relevant difference being found between the control group (*i.e.*, fed with conventional diet) and the others. At the end of the four weeks rats were sacrificed and a pool of clinical chemistry parameters analyzed. Results are summarized in Table 2. Comparison with the literature is not straightforward due to difference in detection techniques, animal age or feeding, among other variables [40]. Thus, according to the Taconic Technical Library, the mean reference value for cholesterol is 2.1 mmol/L for male and 2.4 mmol/L for female, whereas Boehm *et al.* report 1.5 mmol/L for both male and female [40]. This discrepancy is found in almost any of the chemical analytes compared and remarks the importance of control animals to evaluate the course of the experiment. The reference value of our control group (*i.e.*, animals fed with conventional diet) was 1.93 mmol/L, which is within the reported values and closer to that of the Taconic Technical Library [40].

**Table 2:** Clinical chemistry analysis. Mean values and standard deviation from the five animals comprising each group. Those parameters whose differences with the control group are statistically significant are shown in bold and the resulting p-values in brackets. Animals were fed with conventional diet (control), conventional diet supplemented with 2% (w/w) CHOL (diet), or with diet for two weeks and two extra weeks with diet supplemented with 5% cholestyramine or polymers **8** (l-CS), **11** (l-CX) and **21** (l-S_BPY); l = low DVS:saccharide stoichiometry, C = β-CD, X = dextrin, = starch; BPY = bis(2-hydroxyethyl)amine.

	CHOL (mmol/L)	TG (mmol/L)	Creatinine (µmol/L)	AST (U/L)	ALT (U/L)	GGT (U/L)	Glucose (mmol/L)
Control	1.93 ± 0.22	0.73 ± 0.15	51.27 ± 4.42	113.2 ± 26.8	52.4 ± 4.72	0.86 ± 1.23	12.17 ± 3.98
Diet	2.71 ± 0.54 (*p* < 0.02)	1.25 ± 0.23 (*p* < 0.01)	63.65 ± 7.96 (*p* < 0.05)	156.0 ± 72.89	60.0 ± 9.06	0.99 ± 1.58	9.31 ± 3.34
Cholestyramine	1.8 ± 0.32	1.98 ± 0.83 (*p* < 0.02)	64.53 ± 6.19 (*p* < 0.01)	161.2 ± 8.59	74.0 ± 30.18	1.36 ± 1.58	11.01 ± 1.83
Polymer 8	1.75 ± 0.2	0.89 ± 0.23	48.62 ± 6.19	118.4 ± 9.53	53.2 ± 5.02	4.06 ± 3.14	11.2 ± 1.64
Polymer 11	1.61 ± 0.24	0.82 ± 0.22	58.22 ± 9.72	102.4 ± 13.20	41.0 ± 4.95	1.04 ± 0.97	7.64 ± 3.54
Polymer 21	1.64 ± 0.41	0.74 ± 0.19	48.62 ± 4.42	118.2 ± 11.52	55.8 ± 10.38	1.47 ± 1.01	6.07 ± 0.34 (*p* < 0.01)

Frequently hypercholesterolemia is accompanied by increased TG levels and the treatment of hyperlipidemic patients with BAS resins may provoke additional hypertriglyceridemia as the result of the increase of VLDL-TG levels [41]. A detailed analysis of Table 2 reveals that hypercholesterolemia was successfully induced and the values of both CHOL and TG detected in the control group of animals that did not received any compound were 1.4-fold and 1.7 fold, respectively, higher than the group of animals fed with conventional diet, these differences being statistically significant (*p*-value < 0.02 and < 0.01, respectively). Groups whose diet was supplemented with either polymers or cholestyramine showed similar depressed levels of CHOL, but TG in blood were dependent on the treatment. Thus, polymers regularized the TG levels down to the control group fed with conventional diet, whereas cholestyramine induced a 1.6 fold increase. This result is statistically significant (*p*-value < 0.01) and in full agreement with other studies that report that cholestyramine increases the level of TG [42] whereas CDs reduce triglyceridemia [16,18,43].

There exist some controversial results on the toxicity of ingested CD. Thus, while α-CD is undergoing clinical trial for decreasing serum CHOL in humans [23], 5% β-CD has been reported to increase both alanine aminotransferase (ALT) and aspartate aminotransferase (AST) activity and to provoke cell necrosis in rats [18]. The results of the animal experimentation (Table 2) show that the selected polymers do not alter transaminase activity in blood, being the values similar to those of the control group fed with conventional diet except that of gamma-glutamyltransferase (GGT) for polymer **8**. GGT activity has been reported as highly specific for the diagnostic of bile duct cell necrosis [44], but the comparison with the control group did not achieve statistical significance (0.1 < *p*-value < 0.05). The analysis of creatinine (*i.e.*, acute kidney injury) reveals normal values. At this point it is important to recall that cholestyramine yielded the highest creatinine level and that this increase is statistically significant (*p*-value < 0.01). Considering that cholestyramine is a drug currently in use to manage hypercholesterolemia and that its safety has been assayed, the better values of polymers **8**, **11** and **21** suggest that a priori their toxicity does not invalidate them as candidates to manage hypercholesterolemia.

## 3. Experimental Section

### 3.1. Materials

Native β-CD (95%) was bought from TCI Europe N.V. (Zwijndrecht, Belgium) and both dextrin and starch from Fluka (Madrid, Spain). DVS (97%), CHOL (95%), DCA (99%) and CA (98%) were purchased from AlfaAesar (Karlsruhe, Germany). HEPES (4-(2-hydroxyethyl)-1-piperazineethanesulfonic acid), diethylamine, Bis(2-pyridylmethyl)amine and bis(2-hydroxyethyl)amine were acquired from Sigma-Aldrich (Madrid, Spain). Animals and conventional diet were obtained from Harlan (Barcelona, Spain).

### 3.2. Synthesis of a Library of BAS Polymers

#### 3.2.1. Neutral Homo-Polymers 

Starch, dextrin or β-CD (10 g) was dissolved in carbonate buffer (0.5 M, pH 12, 500 mL) under magnetic stirring at room temperature. For the particular case of starch, the complete solubilization required heating to reflux (15–20 min) and then time to reach room temperature. The stirring was continued for 30 min prior to the addition of either 10 or 5 mL of DVS to yield a DVS:Glc molar ratios of 2:1 or 1:1, respectively. The cross-linking reaction was allowed to proceed (7–8 h) resulting in a solid powder that precipitates from the reaction media. The product was isolated by filtration, thoroughly washed with deionized water and then with MeOH and diethyl ether. The resulting cross-linked carbohydrate polymer was dried in vacuo for 18 h at 50 °C.

#### 3.2.2. Neutral Hetero-Polymers 

β-CD (5 g) and starch, dextrin (5 g) were copolymerized with DVS (5 mL, DVS:Glc 1:1 molar ratio). The cross-linking reaction and the isolation of the product was carried out as described above.

#### 3.2.3. Charged Homo- and Hetero-Polymers 

Polymers were prepared as described above. Charge was introduced by addition of 25 mmol of bis(2-pyridylmethyl)amine, bis(2-hydroxyethyl)amine or diethylamine and after 1.5 h of cross-linking reaction. The copolymerization reaction was allowed for 6–7 h prior the isolation of the polymer.

### 3.3. Analysis of the Sequestrant Capability of the Library of BAS Polymers

Polymers were powder and homogenized with a mortar and pestle prior the assay. The polymer (0.5 g) was incubated for 1 h at 37 °C with either 5 mL of a solution of CA (3 mM) and DCA (2 mM) in 25 mM HEPES pH 7.5, 2.5% DMSO (v/v) or with 5 mL of CHOL (5 mM) in THF. After incubation samples were centrifuged at 14000 rpm and the supernatants were analyzed by High Resolution Mass Spectrometry as described in the bibliography [45].

### 3.4. Scale-Up Synthesis of BAS Polymers for Animal Experimentation

The corresponding saccharide or mixture of saccharides (25 g) is dissolved in carbonate buffer (0.5 M pH 12, 2.5 L) under mechanical stirring (700 rpm). The addition of DVS (25 mL) was carried out dropwise and the cross-linking reaction was allowed to proceed overnight. The product was isolated by filtration and thoroughly washed with deionized water, with MeOH and finally with diethyl ether. The resulting cross-linked saccharide polymers were subjected to Soxhlet extraction with ethyl acetate to eliminate any trace of unreacted components and then dried in vacuo for 18 h at 50 °C. Samples were powered with a blender before addition to the diet.

### 3.5. Characterization of the BAS Polymers Assayed in Animal Experimentation

Elemental analyses (CHNS) were determined with an elemental analyzer Thermo Scientific Flash 2000 (Thermo Scientific, Madrid, Spain). Electron microscopy examination was performed on samples covered with gold using a sputter coater (SEMPREP2, Technoorg Linda LTD, Budapest, Hungary) and electron micrographs were taken with a Hitachi S-510C scanning electron microscope at 3 kV (Hitachi High Technologies Europe GmbH, Krefeld, Germany. X-ray diffraction was carried out on a Philips Pw1710/00 diffractometer using Cu Kα radiation (Madrid, Spain). The operation voltage and current were 40 kV and 40 mA, respectively. Data were collected from 3° to 80° with a 0.04° step and 0.4 s of integration. Data acquisition and processing were carried out with Xpowder diffraction software [46].

### 3.6. Animal Experimentation

All animal experiments were carried out in strict compliance with the recommendations in the Guide for the Care and Use of Laboratory Animals of the National Institutes of Health. Animal care and experimental procedures were approved by the *Animal Experimentation Ethics Committee* (CEEA) of the University of Granada, Spain (Ref. 420-2012-CEEA).

A total of 30 male Wistar rats weighing 100 g were randomly divided into 6 groups and they were given *ad libitum* access to food and water. The control group was fed exclusively with a conventional diet. The remaining five groups were fed with conventional diet supplemented with 2% (w/w) cholesterol for two weeks to induce hypercholesterolemia and then for two extra weeks with the same diet supplemented with 5% (w/w) of BAS polymer to assay. At the end of the experimental period animals were sacrificed by cervical dislocation and blood sample were taken for the analysis of cholesterol, triglycerides, glucose, creatinine and transaminases. Clinical Chemistry analyses were carried out by a certified veterinary laboratory.

## 4. Conclusions

The cross-linking of either β-CD, dextrin or starch with DVS yielded a library of insoluble homo- and hetero-polymers. Among the 22 polymer synthesized, three were identified as promising materials for their ability to trap CHOL and BA as well as for the yield of the synthesis. It is important to recall that two are neutral hetero-polymers of β-CD and starch (polymer **8**) or dextrin (polymer **11**) and the third is a weakly cationic homo-polymer of starch functionalized with bis(2-pyridylmethyl)amine, highlighting the importance of the cavity effect. *In vivo* assay on hypercholesterolemic rats reveals that the selected polymers yield similar reduced levels of CHOL. The analysis of the transaminase activity and the creatinine level does not detect any statistically significant difference compared to the control group, supporting the absence of toxicity. In general, the ability is these three polymers to regulate hypecholesterolemia is similar to that for the reference BAS cholestyramine, but with two additional advantages: (i) they normalize the TG level, and (ii) they do not increase the creatinine level. These results suggest that these polymers are good candidates to manage hypercholesterolemia. 
